# Co-Ordination of Local Health Services

**Published:** 1924-03

**Authors:** 


### THE CO-ORDINATION OF LOCAL HEALTH SERVICES.*

### II.—THE POOR LAW UNDER SENTENCE OF DEATH.

CERTAIN specific health functions — a term which may be regarded as embracing personal hygiene and child-welfare—have during the last half-century been added to those already possessed by Poor Law Guardians by the Cleansing of Persons Act, 1897, the Children Act, 1908 (the regulation of baby-farming and the prevention of cruelty to children), and the Vaccination Acts, 1867 to 1907. Apart from these, no new health functions have been conferred upon the Poor Law authorities during a period which has witnessed a considerable development of their health activities. These activities trace their authority to a general requirement, in an old statute of Elizabeth, that the Poor Law authorities should relieve " the lame, impotent, old, blind, and such other among them, being poor, as are not able to work."

### The Abolition of Boards of Guardians.

The Local Government Committee, presided over by Sir Donald Maclean, in its report of December, 1917, recommended the abolition of Boards of Guardians. On the Second Reading of the Bill first introduced for the setting-up of the Ministry of Health, the Minister in charge, Dr. Addison, said that " the Government accept the recommendations of the Committee that all services relating to the care and treatment of the sick and infirm should not be administered as part of the Poor Law, but should be made a part of the general health services of the country. . . . The Government accept the principle that the remaining functions of the Poor Law authorities should also be transferred to other bodies."

### New Rating Proposals.

It is with the services relating to the care and treatment of the sick and infirm that we are here concerned. But, before examining more closely the question of the Poor Law Guardians' health functions, we shall glance briefly at their other functions. They appoint the Overseers who make the assessments throughout the country for the purposes of the Poor Rate. It is of interest to note that under the proposed Bill on rating and valuation, the draft of which has been widely circulated by the Ministry of Health, it is intended to transfer the rating functions of the Overseers to the Town Councils and District Councils, and to substitute boroughs and urban and rural districts for parishes as rating areas. One effect of the change would be to reduce the number of rating authorities from 12,882 to 648 in the rural areas of England and Wales, and from 2,664 to 1,119 in the urban areas. The new Rating Bill seeks to promote uniformity of valuation throughout the country, with one valuation for purposes of local rating and imperial taxation. This will have an important bearing on the general question of co-ordination or inter-relation of medical and sanitary services to which we shall have occasion to refer in a later article.

### The Relief of Distress.

The Guardians' main function, from which spring their activities in regard to the care of the sick poor and the teaching and care of poor children, is the relief of distress. In regard to the unemployed able-bodied, and the assistance to be given in money or in kind to those not actually in need of medical care and treatment, it is clear that the overlapping of the work of the Guardians with that of the other authorities has been aggravated in recent years by the growth of authorities and the multiplication of duties, all concerned with the economic circumstances of those requiring relief. It needs little reflection to see that there are abundant problems for consideration, in regard to the ultimate correlation of Unemployment Insurance authorities, Old Age Pension Committees, and War Pension Committees. The care and feeding of school children is another problem where economic considerations arise.

### The Most Intractable Case of Overlapping.

In regard to the problem as a whole, let us quote what the Maclean Committee said in December, 1917 :—

> We have not attempted in this Report to deal with the whole problem of overlapping local authorities in respect of public assistance. We have limited ourselves to the largest and the most intractable case of overlapping, namely, that of the Boards of Guardians on the one hand, and the County, Municipal and other Health and Education authorities on the other. We are here faced by overlapping functions and areas, and by conflicting principles of administration. The resulting confusion has been aggravated by the growing popular prejudice against the Poor Law—a prejudice which does less than justice to the devoted work of the Guardians and the continuous improvement in poor law administration, especially in respect of the children and the sick. For the last decade Parliament has been unwilling to entrust the Boards of Guardians with new functions, and the provision for new services has had to be made by other local authorities—in some cases new local authorities—often to the increase of the confusion and overlapping. Further, the classification by institutions and the specialised treatment of recipients of assistance almost necessarily involve an enlargement of existing areas of administration. The Royal Commission on the Poor Laws and Relief of Distress, which sat for over three years and took a very large amount of evidence, attached great importance to these points ; and both the Majority and Minority Reports concurred in recommending the abolition of Boards of Guardians and of the Poor Law Union.

### The Poor Law Infirmary Equipment.

It will be seen that testimony is paid by the Committee, in passing, to the devoted work of the Guardians and the continuous improvement in Poor Law administration, especially in respect of the children and the sick. There can be no question about the efficiency of a great many of the Poor Law medical services, especially in many of the larger towns. It is common knowledge that a large and

* This series of articles has been unavoidably delayed. The introductory article appeared in our November number.

increasing number of our Poor Law infirmaries are efficiently equipped and that there are available for the sick poor professional care and equipment too often beyond the reach of those who are of the middle classes. This has led to an increase in demand for accommodation for paying patients in the Poor Law infirmary; and this, in turn, to an outcry against the disability that is involved by the occupation of a bed in a Poor Law institution whereby a patient, notwithstanding that he pays two, three, or four guineas a week, is a pauper as soon as he enters the institution. The Poor Law authorities are, in fact, tending to become general hospital providers, and it will be increasingly difficult to know what distinction to draw between the classes of the community treated and the kind of services rendered in Poor Law institutions—services rendered by a publicly-financed administration—on the one hand, and the classes of the community treated, and the services rendered by our voluntary hospitals.

### Poor Law Medical Developments.

There is not much space given to the question of the Poor Law medical services in Sir George Newman's Report on the state of the public health for 1922, but what is said is very significant:—

The system of appointing medical consultants at Poor Law infirmaries is extending, and in London there are now only three or four infirmaries without one or more consultants. The advantages of such appointments are not confined to the patients, but apply also to the medical and nursing staff as tending to raise the tone of the infirmary. The larger Poor Law infirmaries are, in fact, approximating more and more to the standard of general hospitals. In particular, the great increase of surgical cases and of acute medical cases is noticeable, as also is the considerable number of non-resident and fee-paying patients treated at some infirmaries. While there is no intention of establishing out-patient departments in the ordinary sense, a considerable number of patients are now discharged from the infirmaries at an earlier stage of convalescence than formerly and return at intervals for "continuation treatment." Beds are thus more quickly available for fresh admissions.

### Co-operation Between Guardians and Medical Schools.

And again:—

Advantage has been taken of opportunities which have presented themselves during the year to develop co-operation between Poor Law institutions and medical schools to their mutual advantage. Thus the Bermondsey Board of Guardians have arranged, with the approval of the Minister, that certain of their infirmary wards should be made available for medical education in connection with Guy's Hospital Medical School, and a similar arrangement has been sanctioned at Cardiff, Lambeth and several other centres. Steps have also been taken to secure co-operation between Poor Law institutions and clinics for the treatment of tuberculosis and venereal disease.

### Prevention and Cure.

The case for the abolition of the Guardians rests, so far as their health functions are concerned, not on any suggestion that those functions are inefficiently performed; this is so far from being the case that many of the Poor Law infirmaries with their staffsand equipment would be a real acquisition for the community as a whole. But the existence of a separate and a costly administration for dealing with the health of one section of the community is unsound in principle. Indeed, the primary duty of the Guardians, that of determining the economic condition of the persons applying for treatment as poor persons, should be an incident, a not unimportant one, it is true, of a more widely-conceived health administration. This is recognised in the recommendation of the Maclean Committee that the County and County Borough Councils—to whom the functions of the Guardians should, in their view, be transferred—should set up Home Assistance Committees, free from any suspicion of using the "workhouse test" or the policy of "deterrence," and having the duty of making all necessary enquiries into the economic circumstances of those seeking relief. Further, it is of the essence of any sound scheme for the co-ordination of health services that the general responsibility for the prevention and for the cure of disease should be vested in one authority, so that all measures directed to either or both of these objects should be properly related and directed. In the Poor Law we have an administering authority, levying enormous public funds for the discharge of its functions, which arise from the existence of disease and distress, yet having no duties or powers in respect of their prevention.

